# A Simultaneously Calibration Approach for Installation and Attitude Errors of an INS/GPS/LDS Target Tracker

**DOI:** 10.3390/s150203575

**Published:** 2015-02-04

**Authors:** Jianhua Cheng, Daidai Chen, Xiangyu Sun, Tongda Wang

**Affiliations:** 1 Marine Navigation Research Institute, College of Automation, Harbin Engineering University, Harbin 150001, China; E-Mails: chen.daidai@aalto.fi (D.C.); ins_xiang@163.com (X.S.); wangtongda1990@163.com (T.W.); 2 Department of Biomedical Engineering and Computational Science, School of Science, Aalto University, Espoo 02150, Finland

**Keywords:** INS/GPS/LDS, target tracking, measurement, installation error, calibration

## Abstract

To obtain the absolute position of a target is one of the basic topics for non-cooperated target tracking problems. In this paper, we present a simultaneously calibration method for an Inertial navigation system (INS)/Global position system (GPS)/Laser distance scanner (LDS) integrated system based target positioning approach. The INS/GPS integrated system provides the attitude and position of observer, and LDS offers the distance between the observer and the target. The two most significant errors are taken into jointly consideration and analyzed: (1) the attitude measure error of INS/GPS; (2) the installation error between INS/GPS and LDS subsystems. Consequently, a INS/GPS/LDS based target positioning approach considering these two errors is proposed. In order to improve the performance of this approach, a novel calibration method is designed to simultaneously estimate and compensate these two main errors. Finally, simulations are conducted to access the performance of the proposed target positioning approach and the designed simultaneously calibration method.

## 1. Introduction

To measure the three-dimensional (3D) position of a target in motion is one of the basic problems of target tracking. In this section, we will briefly review the sensors commonly used for measuring the position of a target. Consequently, an Inertial navigation system (INS)/Global position system (GPS)/Laser distance scanner (LDS) integrated system based target positioning approach is introduced.

### 1.1. Sensors for Target Tracking

Target tracking [1] by using different sensors is a significant field of work for the application areas of military applications [2,3], pedestrians surveillance [4,5] and autonomous robotics [6,7]. Most existing research work on target tracking concentrates on efficient ways of modeling [8,9] and trajectory estimation methods [10,11], as well as on varieties of data association approaches[12,13], which are extremely significant for the multiple target tracking problem [14,15].

However, there is comparatively less work that refers to the sensors in target tracking. Meanwhile, only a little of the literature is concerned about the low-cost target tracking field. For the demonstration of a 3D object’s motion, the parameters related to the linear motion and rotation is extremely essential information.

For the tracking problem of cooperated targets, generally almost all the navigation systems and their sensors are efficient information sources, such as INS, GPS and celestial navigation system. These traditional navigators based on different positioning principles could supply the 3D position. However, it is necessary to mount these navigators and sensors on the targets, which is practically impossible when tracking a non-cooperative target.

For the sensors commonly used in target tracking, we put forward a summary as follows. In this summary, some special sensitive sensors are not included, such as the gravity instrument and magnetometer.

(1) Radar [16,17]—which might be the most predominant sensor in this field, and its advanced developments, such as the monostatic radar and multistatic radar (bistatic radar, multi-input and multi-output radar [18]). A radar exhibits excellent performance for detection and feedback measurements, not only the range information of targets but also their bearings.
(2) IR sensor [19–21]—which is often used to detect and locate the infra-red emit object by visual, or performs as a thermal imaging system to provide a radiant temperature distribution graph. Individually or as an assisted subsystem, the IR sensor has be widely used in military applications, such as the airplane forward/side looking system [22] and the detector for smart attack munition.
(3) Electronic-optic sensor [23–25]—which is used for the both-way converts between light and electronic signals, typically the camera.
(4) Electronic support measurement [15]—could only provide angular measurements, and usually passively detecting the radar signals delivered by targets.
(5) Laser sensor [26,27]—which is ideal for the non-contact measurement of range, with a long effective range. A detection method based on the laser scanner could obtain the 3D position of the surface of objects by laser distance detecting. However, the obtained 3D position are the information the object corresponding to the instrument coordinates.
(6) Acoustic sensor [28,29]—which could convert the physical acoustic signals into digital signals, specially suitable for the underwater vehicle detection and tracking, despite easily be influenced by environment noise. Sonar is a common one for this kind sensor.
(7) Sensor network [30,31]—which commonly utilize multiple or even multiclass low-accuracy and cheap sensors to efficiently tracking for randomly appearing objects. Their effective range is usually small, and they are frequently used in the indoor or local area surveillance. Wireless sensor network [28] are developed to extend the range as well as reduce the complex of physical network connection.
(8) Binary proximity sensor [32,33]—which is low-cost and economical for the computation complexity for only provide binary detection, usually combined to form sensor network.

### 1.2. INS/GPS/LDS System Based 3D Positioning Approach

The mapping principle of 3D laser scanner [27], which is quite similar to that of the optical theodolite and the total station device, is based on the characteristic of straight propagation of the light. The only difference is that 3D laser scanner has one more function that could measure the distance between the device and its target.

By this mapping method, we could obtain the 3D coordinates of the point at the surface of target, and then quickly establish a 3D model of the object. However, this method has a disadvantage that the obtained coordinates of the points are related to the measurement coordinate system [34]. In [35], a method is designed for target navigation and mapping based on the INS/GPS/LDS integrated system, in which inertial sensors are utilized to provide the rotation measurements to map the range information in the measurement coordinate system into that in the geographic coordinate. The impact of the attitude errors of inertial sensors on the mapping performance are consequently investigated and calibrated. With a high-frequency update, this method could be used for target tracking. However, the work of [35] is based on an assumption that all the three subsystems are precisely installed. Before the real application, the misalignment error between the sensitive axes of INS/GPS system and LDS should be compensated to be minimum, to avoid an over large positioning error.

Suffering from the limits of machining and the measurement error of devices, there are non-orthogonal coordinate systems that exist in the INS/GPS/LDS system:

(1) the non-orthogonal coordinate of inertial sensors;
(2) the non-orthogonal coordinate between LDS and INS/GPS system.

To guarantee the accuracy of measurement, calibration is needed to transform the non-orthogonal coordinates into the orthogonal ones. For instance, for a INS/GPS integrated system, the transformation can be realized by conducting the calibration by using a three-axle rotational table in the laboratory [36,37]. As for the laser scanner, [38,39] give some suitable calibration schemes. However, the content of calibration for the misalignment between INS/GPS and LDS is almost blank for the different measurement principles of these two systems. Advanced physical machining and precisely installation seem like available solutions, but undoubtedly they will lead to the problem of high cost and strict demands for the machining technology.

In this paper, we present an error calibration method for an INS/GPS/LDS tracker, to jointly estimate and compensate the installation and attitude errors. In Section 2, the target positioning method based on INS/GPS/LDS is analyzed and then a modified target mapping approach by considering the installation error between INS/GPS and LDS subsystems is presented. In Section 3, the target positioning error of this approach is investigated by taking the installation error into consideration. Furthermore, we address the corresponding calibration algorithm in Section 4, in which external reference points are introduced and their information are adopted to synchronously calibrate both the installation error and the attitude measurement error of INS/GPS system. Finally, simulations are conducted to verify the validity of the calibration algorithm and the positioning performance of the entire system.

## 2. Algorithm Description for Calculating the Position of Target

### 2.1. The Conventional INS/GPS/LDS System Based Approach

**Figure 1.**
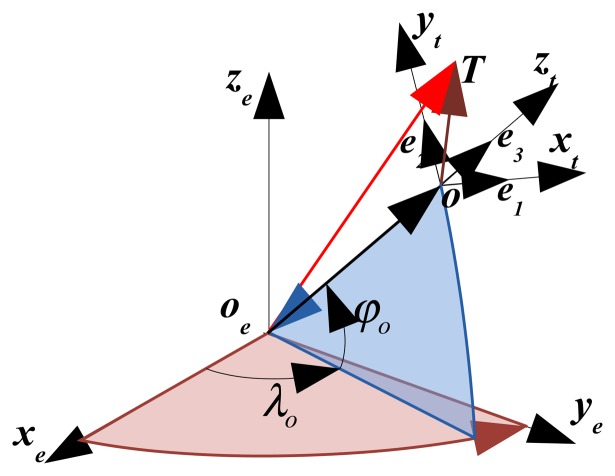
Description of *T* in the geographic and the earth coordinate system.

For the convenience of calculating the target coordinate with the conventional INS/GPS/LDS system based method, a vector of **oT** defined between the observation point o to the target *T* is introduced. The mathematical representation of **oT** is related with distance *d*, yaw *H* and pitch *ϕ* (the angles are defined in the local geographic coordinate system) between o and *T*. In order to calculate the absolute coordinate of *T*, the vector **oT** should be converted into a corresponding vector within the earth coordinate system. In this convert, the necessary information we needed includes: (a) the position information of the target *T*, which has been measured in the local geography coordinate system; (b) the position of the observation point *o* in the earth coordinate system. Figure 1 shows the target *T* in the geographic *ox_t_y_t_z_t_* and earth coordinate system *ox_e_y_e_z_e_*.

The basic procedure of the INS/GPS/LDS based approach are

(1) Definition of the basis vector **oC** in *ox_t_y_t_z_t_*
  In order to demonstrate **oT** by using the attitudes defined in *ox_t_y_t_z_t_*, which are measured by INS/GPS subsystem, a basis vector of **oC** is defined along the *oy_t_* axis of *ox_t_y_t_z_t_*. The vector is described as
  (1) $$\text{oC}=0{\text{e}}_{1}+d{\text{e}}_{2}+0{\text{e}}_{3}$$
  where, e_1_, e_2_, e_3_ are the unit vectors along the three axes of *ox_t_y_t_z_t_*, respectively; and *d* is the distance between *o* and *T*.
(2) Describe the vector of **oT** in *ox_t_y_t_z_t_*
  The measure principle of LDS guarantee its sensitive axis would have the same direction with the vector of **oT**. Therefore, we assume that the direction of **oT** is along with the *oy_b_* axis of the body coordinate system *ox_b_y_b_z_b_*. The vector **oT** can obtained from **oC** after twice rotations with the angles of *H* and ϕ, respectively. The rotations can be expressed by the direction cosine matrix as
  (2) Ctb=[1000cosϕsinϕ0−sinϕcosϕ]⋅[cosHsinH0−sinHcosH0001]
  where, ϕ and *H* denote the pitch and yaw, respectively. The angles and the transform matrix ${\text{C}}_{t}^{b}$ are used to describe the twice rotation relationship between the geographic *ox_t_y_t_z_t_* and the body coordinate system *ox_b_y_b_z_b_*, where is the platform LDS placed. Then, the vector **oT** can be described in the geographic coordinate system:
  (3) $$\text{oT}={\text{C}}_{t}^{b}\cdot \text{oC}$$
  The 3D coordinate of *o* in *ox_t_y_t_z_t_* can be obtained by combining Equations (2) and (3) as follows.
  (4) $$\left[\begin{array}{l} x_{oT}^{t}\\ y_{oT}^{t}\\ z_{oT}^{t} \end{array}\right]=\left[\begin{array}{ccc} 1 & 0 & 0\\ 0 & \cos\phi & \sin\phi \\ 0 & -\sin\phi & \cos\phi \end{array}\right]\cdot \left[\begin{array}{ccc} \cos H & \sin H & 0\\ -\sin H & \cos H & 0\\ 0 & 0 & 1 \end{array}\right]\cdot \left[\begin{array}{c} 0\\ d\\ 0 \end{array}\right]=\left[\begin{array}{c} d\sin H\\ d\cos\phi \cos H\\ -d\sin\phi \cos H \end{array}\right]$$
(3) Describe the vector **oT** in *ox_e_y_e_z_e_*
  Provided the latitude *φ_o_* and longitude λ*_o_* of the point *o*, the 3D coordinate of **oT** after being converted from *ox_t_y_t_z_t_* to *ox_e_y_e_z_e_* is
  (5) $$\left[\begin{array}{c} x_{oT}^{e}\\ y_{oT}^{e}\\ z_{oT}^{e} \end{array}\right]={\text{C}}_{t}^{e}\cdot \left[\begin{array}{c} x_{oT}^{t}\\ y_{oT}^{t}\\ z_{oT}^{t} \end{array}\right]$$
  where, ${\text{C}}_{t}^{e}$ is the transform matrix from the geographic coordinate system *ox_t_y_t_z_t_* to the earth coordinate system *ox_e_y_e_z_e_* [40], and
  (6) $${\text{C}}_{t}^{e}=\left[\begin{array}{ccc} \cos\lambda_{o} & -\sin\lambda_{o}\cos\varphi_{o} & \sin\lambda_{o}\cos\varphi_{o}\\ \cos\lambda_{o} & \cos\lambda_{o}\cos\varphi_{o} & -\cos\lambda_{o}\sin\varphi_{o}\\ 0 & \sin\varphi & \cos\varphi_{o} \end{array}\right]$$

As shown in Figure 1, three vectors **o**_e_**T**, **o**_e_**o** and **oT** follow the relationship:

(7) $${\text{o}}_{e}\text{T}={\text{o}}_{e}\text{o}+\text{oT}$$

By Equations (5) and (7), the 3D absolute position of target in *ox_e_y_e_z_e_* can be calculated:

(8) $$\left[\begin{array}{l} x_{T}^{e}\\ y_{T}^{e}\\ z_{T}^{e} \end{array}\right]=\left[\begin{array}{l} x_{o}^{e}\\ y_{o}^{e}\\ z_{o}^{e} \end{array}\right]+{\text{C}}_{t}^{e}\;\left[\begin{array}{c} d\sin H\\ d\cos\phi \sin H\\ -d\sin\phi \cos H \end{array}\right]$$

where, ${\left[x_{T}^{e},y_{T}^{e},z_{T}^{e}\right]}^{T}$ and ${\left[x_{o}^{e},y_{o}^{e},z_{o}^{e}\right]}^{T}$ are the 3D coordinates of target *T* and the observer point *o* in the earth coordinate system *ox_e_y_e_z_e_*, respectively. The geographic coordinate of the observer point (latitude *φ_o_* and longitude λ*_o_*), and the 3D coordinate $\left[x_{o}^{e},y_{o}^{e},z_{o}^{e}\right]$, the yaw *H*, the pitch ϕ can be measured by INS/GPS subsystem, while the distance *d* between the target and the observer point can be measured by LDS. As seen from Equation (8), it is obvious that the absolute 3D coordinate of the target can be calculated by the measurements of INS/GPS/LDS. In addition, other useful information such as the height difference, incline angle, horizontal angle can also be introduced to assist the calculation.

### 2.2. Problems Caused by the Initial Installation Error

According to the previous analysis in [35], each part of INS/GPS/LDS integrated system would inevitably have measurement error. Therefore, the actual coordinate should be expressed as:

(9) $$\left[\begin{array}{l} {\tilde{x}}_{T}^{e}\\ {ỹ}_{T}^{e}\\ {\tilde{z}}_{T}^{e} \end{array}\right]=\left[\begin{array}{l} {\tilde{x}}_{o}^{e}\\ {ỹ}_{o}^{e}\\ {\tilde{z}}_{o}^{e} \end{array}\right]+{\text{C}}_{t}^{e}\;\left[\begin{array}{c} \tilde{d}\sin\tilde{H}\\ \tilde{d}\cos\tilde{\phi }\sin\tilde{H}\\ -\tilde{d}\sin\tilde{\phi }\cos\tilde{H} \end{array}\right]$$

where, the variables with the superscript “∼” denote that their values contain errors. In [35], among the error sources, the attitude error of INS/GPS dominates the calculation accuracy. By introducing an outer reference point, the yaw error and pitch error of INS/GPS can be precisely estimated and compensated.

However, besides the attitude error, the initial installation error between INS/GPS and LDS subsystems should be also taken into consideration. The sensitive axes of these two systems should be maintained along the same directions, or aligned with the corresponding installation basis. Otherwise, the angle difference between the vectors **oT** and **oC** should not be equal to the yaw and pitch outputted by INS/GPS system.

To calibrate the installation error, physical calibration methods are commonly adopted among many engineering applications. However, such kinds of solutions usually demand high-accuracy instruments, which could provide precise reference for installation, leading to over high requirement for machining as well as the cost. Based on the experience of calibration for the attitude error of INS/GPS in [35], soft calibration schemes appear to be more reasonable. It seems also available to estimate both the installation and attitude error by using the information of other reference points, which have been already precisely measured.

### 2.3. Target Positioning Algorithm by Considering the Installation Error

In the general inertial measurement unit, each gyro and its corresponding accelerometer should be aligned with the same direction. Unfortunately, it is almost impossible to realize that. Consequently, two non-orthogonal coordinate systems are formed, which are consisting of gyroscopes and accelerometers, respectively. Calibration for these installation error and non-orthogonal characteristics is necessary for their effective operation. The two non-orthogonal coordinate systems should be converted into orthogonal coordinate systems [37], which is also needed for the LDS and INS/GPS.

**Figure 2.**
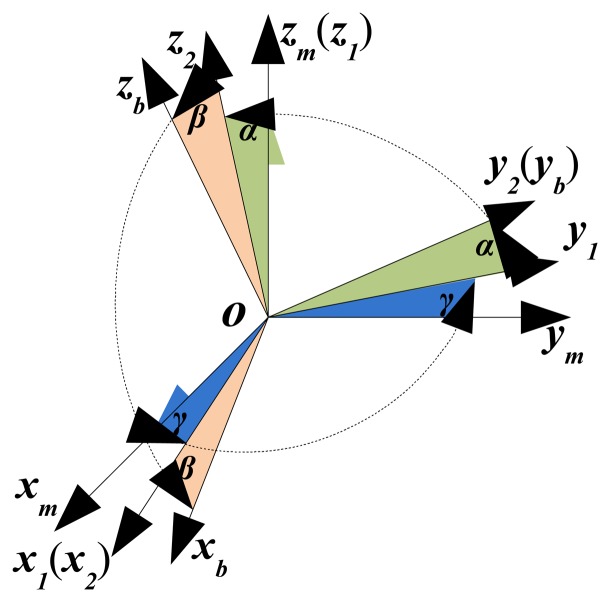
Rotation relationship between *ox_m_y_m_z_m_* and *ox_b_y_b_z_b_*.

Assuming that the calibrated coordinate system of INS/GPS is *ox_m_y_m_z_m_*, LDS is mounted along the axis of *oy_b_*. There are three misalignment angles *α, β* and *γ* between *ox_m_y_m_z_m_* and *ox_b_y_b_z_b_*, which denote the installation error between INS/GPS and LDS subsystems, as shown in Figure 2. Considering the minor angle situation, the transformation matrix between the two coordinate systems of INS/GPS and LDS can be expressed as:

(10) $${\text{C}}_{m}^{b}=\left[\begin{array}{ccc} 1 & \gamma & -\beta \\ -\gamma & 1 & \alpha \\ \beta & -\alpha & 1 \end{array}\right]$$

As can be seen in Figure 2, when the installation error between INS/GPS and LDS exists, the yaw and pitch outputs of INS/GPS are not equal to the rotation angles of **oC**. The main reason is that only LDS is mounted along the axis *oy_b_*, which is fixed in *ox_b_y_b_z_b_*. However, for the rotation defined in the measurement coordinate system of INS/GPS, the actual rotational angle relates to the axis *oy_m_* .

Define a new vector **oC***_m_*, whose mode is *d* and its direction along with the axis **oy***_m_*. Therefore, the vector **oC***_m_* can be expressed as

(11) $$\text{o}{\text{C}}_{m}={\text{C}}_{b}^{m}\cdot \text{oC}$$

where, ${\text{C}}_{b}^{m}={\left({\text{C}}_{b}^{m}\right)}^{T}$.

It can be seen from Equation (11) that the rotation described by the attitude measured by INS/GPS can be a vector rotated by **oC***_m_*, which could indicate the actual target position impacted by the misalignment angles or installation error.

Substituting Equation (11) into Equation (9), we could obtain:

(12) $$\left[\begin{array}{c} {\tilde{x}}_{T}^{e}\\ {ỹ}_{T}^{e}\\ {\tilde{z}}_{T}^{e} \end{array}\right]=\left[\begin{array}{c} {\tilde{x}}_{o}^{e}\\ {ỹ}_{o}^{e}\\ {\tilde{z}}_{o}^{e} \end{array}\right]+{\text{C}}_{t}^{e}\cdot {\text{C}}_{t}^{b}\;\left[\begin{array}{ccc} 1 & \gamma & -\beta \\ -\gamma & 1 & \alpha \\ \beta & -\alpha & 1 \end{array}\right]\cdot \left[\begin{array}{c} 0\\ \tilde{d}\\ 0 \end{array}\right]$$

After simplification, the target positioning algorithm by considering the installation error can be expressed as:

(13) $$\left[\begin{array}{c} {\tilde{x}}_{T}^{e}\\ {ỹ}_{T}^{e}\\ {\tilde{z}}_{T}^{e} \end{array}\right]=\left[\begin{array}{c} {\tilde{x}}_{o}^{e}\\ {ỹ}_{o}^{e}\\ {\tilde{z}}_{o}^{e} \end{array}\right]+{\text{C}}_{t}^{e}\;\left[\begin{array}{c} \tilde{d}\sin\tilde{H}-\gamma \tilde{d}\cos\tilde{H}\\ \tilde{d}\cos\tilde{\phi }\cos\tilde{H}+\gamma \tilde{d}\cos\tilde{\phi }\sin\tilde{H}+\alpha \tilde{d}\sin\tilde{\phi }\\ -\tilde{d}\cos\tilde{\phi }\cos\tilde{H}-\gamma \tilde{d}\sin\tilde{\phi }\sin\tilde{H}+\alpha \tilde{d}\cos\tilde{\phi } \end{array}\right]$$

Obviously, the installation error denoted by two misalignment angles of ϕ and *γ* will directly influence the 3D coordinate’s calculation.

## 3. Positioning Error Analysis by Considering Installation Error

Under an ideal condition without installation error, a positioning error analysis has been conducted in [35]. As its conclusion, the attitude error of INS/GPS appears to be the most dominant error source. In this section, the extended error analysis about the installation error are implemented.

By ignoring the other errors of INS/GPS and LDS, the positioning error caused by non-ideal installation can be obtained by the minus between Equations (13) and (8), which is given as:.

(14) $$\Delta_{T}=\left[\begin{array}{c} {\tilde{x}}_{T}^{e}\\ {ỹ}_{T}^{e}\\ {\tilde{z}}_{T}^{e} \end{array}\right]-\left[\begin{array}{c} x_{T}^{e}\\ y_{T}^{e}\\ z_{T}^{e} \end{array}\right]={\text{C}}_{t}^{e}\;\left[\begin{array}{c} -\gamma d\cos H\\ \gamma d\cos\phi \sin H+\alpha d\sin\phi \\ -\gamma d\sin\phi \sin H+\alpha d\cos\phi \end{array}\right]$$

where, $\Delta_{T}={\left[\Delta x_{T}^{e},\Delta y_{T}^{e},\Delta z_{T}^{e}\right]}^{T}$ is the positioning error caused by the installation error. The matrix of ${\text{C}}_{t}^{e}$ in Equation (14) is an orthogonal matrix, which exhibits the following properties:

(15) $$\{\begin{array}{l} {\left({\text{C}}_{t}^{e}\right)}^{-1}={\left({\text{C}}_{t}^{e}\right)}^{T}\\ \left|{\text{C}}_{t}^{e}\right|=1. \end{array}$$

The variance of positioning error caused by the installation error can be calculated:

(16) $$\Delta P_{T}=\sqrt{{\left(\Delta_{T}\right)}^{T}\Delta_{T}}=\sqrt{{\left(\Delta_{\phi }\right)}^{T}\cdot {\left({\text{C}}_{t}^{e}\right)}^{T}\cdot \Delta_{\phi }}$$

where,

$$\Delta_{\phi }=\left[\begin{array}{c} -\gamma d\cos H\\ \gamma d\cos\phi \sin H+\alpha d\sin\phi \\ -\gamma d\sin\phi \sin H+\alpha d\cos\phi \end{array}\right]$$

Substituting Equations (14) and (15) into Equation (16), we obtain

(17) $$\Delta P_{T}=\sqrt{{\left(\Delta_{T}\right)}^{T}\Delta_{T}}=d\cdot \sqrt{{\left(\phi \right)}^{2}+{\left(\gamma \right)}^{2}}$$

As for Equation (17), the minor error angle condition is taken into consideration. The term $\sqrt{{\left(\phi \right)}^{2}+{\left(\gamma \right)}^{2}}$ denotes their numerical solution.

In the following part, simulations are conducted to provide the mathematical explain for the analysis result shown in Equation (17). Assuming the initial position of the point *o* is obtained (with the longitude 120° and the latitude 45°), and the measure noise for position is 0.2 m; the yaw and pitch error of INS/GPS is 0.2° and 0.1°, respectively. The measure noise of LDS is 5 mm for the target within 300 m, and has an additional linearity error for 0.15% for the target out of the range of 300 m [41]. The same swing manner of target is set to follow the ideal yaw *H* = 30° + 7° sin(2*π* × *t*/7) and the pitch *ϕ* = 2° + 1° sin(2*π* × *t*/7).

Condition 1: only the measure errors of INS/GPS and LDS are involved;
Condition 2: only the installation error of *α* = *γ* = −0.1° are involved;
Condition 3: only the installation error of *α* = *γ* = −0.2° are involved;
Condition 4: the measure error of LDS, INS/GPS and the installation error of *α* = *γ* = −0.2° are both involved.

**Figure 3.**
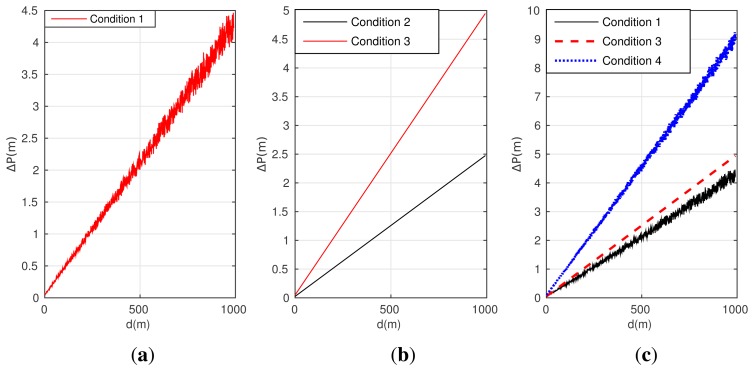
Positioning error simulated results: (**a**) Error of condition 1; (**b**) Error of condition 2 and 3; (**c**) Error of condition 1, 3 and 4.

Figure 3 gives the simulation result of the positioning error corresponding to these four conditions.

As shown in Figure 3a, a quite large positioning error will be excited by the un-calibrated attitude error of INS/GPS, and the value of positioning error increases with the distance between the observer point and the target. Figure 3b shows that the installation error can also excite a large positioning error, and the error by condition 3 is larger than condition 2, so the misalignment angles should be compensated for as closely as possible. From Figure 3c, both two kinds of errors jointly excite obviously larger positioning error than condition 1 and 3, with near 9 meters positioning error at the distance of 1 km.

According to the simulation results in Figure 3, compared with the attitude error of INS/GPS, the installation error between INS/GPS and LDS exhibits almost equivalent influence on the target position error. As a result, these two errors should be compensated for together.

## 4. Simultaneous Calibration Algorithm

By introducing the information of reference point, we can compensate the measurement error of attitudes [35]. In this section, we address the simultaneous calibration method for these two errors.

It should be noticed that LDS could provide high accurate distance measurement especially within a range of 300 m, such as DISTO D810 of LEICA [42], GLM 80 of BOSCH [43], PL-1 of HILTI [44]. The ranging errors of these three products are less than 10 mm. Therefore, once the reference points fall within the range of 300 m, it would definitely guarantee the accuracy of reference information, which will benefit the calibration.

When we neglect the positioning error of INS/GPS and the ranging error of LDS in Equation (13), the real observation equation of the reference point *R* can be obtained:

(18) $$\left[\begin{array}{c} {\tilde{x}}_{R}^{e}\\ {ỹ}_{R}^{e}\\ {\tilde{z}}_{R}^{e} \end{array}\right]=\left[\begin{array}{c} x_{o}^{e}\\ y_{o}^{e}\\ z_{o}^{e} \end{array}\right]+{\text{C}}_{t}^{e}\cdot \left[\begin{array}{c} d_{oR}\sin\tilde{H}-\gamma d_{oR}\cos\tilde{H}\\ d_{oR}\cos\tilde{\phi }\cos\tilde{H}+\gamma d_{oR}\cos\tilde{\phi }\sin\tilde{H}+\alpha d_{oR}\sin\tilde{\phi }\\ -d_{oR}\cos\tilde{\phi }\cos\tilde{H}-\gamma d_{oR}\sin\tilde{\phi }\sin\tilde{H}+\alpha d_{oR}\cos\tilde{\phi } \end{array}\right]$$

where, ${\left[{\tilde{x}}_{R}^{e},{ỹ}_{R}^{e},{\tilde{z}}_{R}^{e}\right]}^{T}$ denotes the measurement of the point *R*, ${\left[x_{o}^{e},y_{o}^{e},z_{o}^{e}\right]}^{T}$ denotes the position of the point *o*, On the other side, the ideal position of the reference point *R* can be expressed as:

(19) $$\left[\begin{array}{l} x_{R}^{e}\\ y_{R}^{e}\\ z_{R}^{e} \end{array}\right]=\left[\begin{array}{l} x_{o}^{e}\\ y_{o}^{e}\\ z_{o}^{e} \end{array}\right]+{\text{C}}_{t}^{e}\cdot \left[\begin{array}{c} d_{oR}\sin H\\ d_{oR}\cos\phi \sin H\\ -d_{oR}\sin\phi \cos H \end{array}\right]$$

From Equations (18) and (19), the measurement error of the point *R* can be calculated.

(20) $$\left[\begin{array}{c} \Delta x_{R}^{e}\\ \Delta y_{R}^{e}\\ \Delta z_{R}^{e} \end{array}\right]=\left[\begin{array}{c} {\tilde{x}}_{R}^{e}-x_{R}^{e}\\ {ỹ}_{o}^{e}-y_{R}^{e}\\ {\tilde{z}}_{o}^{e}-z_{R}^{e} \end{array}\right]={\text{C}}_{t}^{e}\cdot \left[\begin{array}{c} d_{oR}\left(\sin\tilde{H}-\gamma \sin\tilde{H}-\sin H\right)\\ d_{oR}\left(\cos\tilde{\phi }\cos\tilde{H}+\gamma \cos\tilde{\phi }\sin\tilde{H}+\alpha \sin\tilde{\phi }-\cos\phi \cos H\right)\\ -d_{oR}\left(\sin\tilde{\phi }\cos\tilde{H})+\gamma \sin\tilde{\phi }\sin\tilde{H}+\alpha \cos\tilde{\phi }-\sin\phi \cos H\right) \end{array}\right]$$

where, ${\left[\Delta x_{R}^{e},\Delta y_{R}^{e},\Delta z_{R}^{e}\right]}^{T}$ denotes the measurement error of the point *R*.

With Equation (20), both the installation error and the attitude measure error can be estimated with the measurement error of the point *R*, which can be measured by the designed target positioning algorithm and INS/GPS. However, there are two problems that need to be solved:

(1) The Equation (20) is a transcendental equation which could only feedback an approximate analytical solution;
(2) Three observation equations derived from Equation (20) cannot calculate all the four unknown variables of *α, γ, H* and ϕ.

For the first problem, we tend to make some reasonable replacement of the parameters to transform the transcendental equation into an linear equation. Considering that the attitude measure error of INS/GPS are minor angles, we have

(21) $$\{\begin{array}{l} \phi =\tilde{\phi }-\Delta \phi \\ H=\tilde{H}-\Delta H \end{array}$$

where, Δ*ϕ* and Δ*H* are the yaw error and pitch error of INS/GPS, respectively.

Substituting Equations (21) into (20), the transcendental equation can be converted into the following linear form.

(22) $$\left[\begin{array}{c} \Delta x_{R}^{e}\\ \Delta y_{R}^{e}\\ \Delta z_{R}^{e} \end{array}\right]={\text{C}}_{t}^{e}\cdot \left[\begin{array}{c} d_{oR}\left(\Delta H\cos\tilde{H}-\gamma \cos\tilde{H}\right)\\ -d_{oR}\left(\Delta \phi \sin\tilde{\phi }\cos\tilde{H}+\Delta H\cos\tilde{\phi }\sin\tilde{H}-\gamma \cos\tilde{\phi }\sin\tilde{H}-\alpha \sin\tilde{\phi }\right)\\ -d_{oR}\left(\Delta \phi \cos\tilde{\phi }\cos\tilde{H}-\Delta H\sin\tilde{\phi }\sin\tilde{H}+\gamma \sin\tilde{\phi }\sin\tilde{H}-\alpha \cos\tilde{\phi }\right) \end{array}\right]$$

Then, Equation (22) can be rewritten as the matrix style

(23) $$\left[\begin{array}{c} \Delta x_{R}^{e}\\ \Delta y_{R}^{e}\\ \Delta z_{R}^{e} \end{array}\right]={\text{C}}_{t}^{e}\cdot \left[\begin{array}{cccc} 0 & d_{oR}\cos\tilde{H} & 0 & -d_{oR}\cos\tilde{H}\\ -d_{oR}\sin\tilde{\phi }\cos\tilde{H} & -d_{oR}\cos\tilde{\phi }\sin\tilde{H} & d_{oR}\sin\tilde{\phi } & d_{oR}\cos\tilde{\phi }\sin\tilde{H}\\ -d_{oR}\cos\tilde{\phi }\cos\tilde{H} & -d_{oR}\sin\tilde{\phi }\sin\tilde{H} & d_{oR}\cos\tilde{\phi } & -d_{oR}\sin\tilde{\phi }\sin\tilde{H} \end{array}\right]\cdot \left[\begin{array}{c} \Delta \phi \\ \Delta H\\ \alpha \\ \gamma \end{array}\right]$$

It is noted that the second and fourth row of the matrix in Equation (23) are equivalent and just exhibit opposite signs. That means Δ*H* and *γ* can not be calculated separately. Thus, the observation equation should be transformed to the new style with a combined parameter by Δ*H* and *γ*.

(24) $$\left[\begin{array}{c} \Delta x_{R}^{e}\\ \Delta y_{R}^{e}\\ \Delta z_{R}^{e} \end{array}\right]={\text{C}}_{t}^{e}\cdot \text{C}\cdot \;\left[\begin{array}{c} \Delta \phi \\ \Delta H-\gamma \\ \alpha \end{array}\right]$$

where, $\text{C}=\left[\begin{array}{ccc} 0 & d_{oR}\cos\tilde{H} & 0\\ -d_{oR}\sin\tilde{\phi }\cos\tilde{H} & -d_{oR}\cos\tilde{\phi }\sin\tilde{H} & d_{oR}\sin\tilde{\phi }\\ -d_{oR}\cos\tilde{\phi }\cos\tilde{H} & -d_{oR}\sin\tilde{\phi }\sin\tilde{H} & d_{oR}\cos\tilde{\phi } \end{array}\right]$.

Unfortunately, a new problem comes out, that is *det*|**C**| = 0. So the matrix **C** is irreversible, which make the calculation of the four unknown variables impossible. The problem of *det*|**C**| = 0 and the second problem still make the attitude error and misalignment angles can not be uncalculated.

However, if we could find two known reference points of *R*_1_ and *R*_2_, then two observation matrices (or six observation equations) can be established, making the calculation of the installation and the attitude error available. For the convenience of calculation, Equation (24) need to be further simplified. The final observation equation can be obtained by left multiple matrix ${\left({\text{C}}_{t}^{e}\right)}^{-1}$ on both sides of Equation (24).

(25) $$\text{Z}=\text{C}\;\cdot \;\left[\begin{array}{c} \Delta \phi \\ \Delta H_{\gamma }\\ \alpha \end{array}\right]$$

where, Δ*H_γ_* = Δ*H* − *γ*, $\text{Z}=\left[\begin{array}{c} \cos\lambda_{o}\Delta x_{R}^{e}+\sin\lambda_{o}\Delta y_{R}^{e}\\ -\sin\lambda_{o}\cos\varphi_{o}\Delta x_{R}^{e}+\cos\lambda_{o}\cos\varphi_{o}\Delta y_{R}^{e}+\sin\lambda_{o}\Delta z_{R}^{e}\\ \sin\lambda_{o}\sin\varphi_{o}\Delta x_{R}^{e}-\cos\lambda_{o}\sin\varphi_{o}\Delta y_{R}^{e}+\cos\lambda_{o}\Delta z_{R}^{e} \end{array}\right]$.

Provided two reference points, two observation variables **Z**_1_, **Z**_2_, and two corresponding observation matrices **C**_1_, **C**_2_ can be used to construct the joint observation equation:

(26) $$\left[\begin{array}{c} {\text{Z}}_{1}\\ {\text{Z}}_{2} \end{array}\right]=\left[\begin{array}{c} {\text{C}}_{1}\\ {\text{C}}_{2} \end{array}\right]\cdot \left[\begin{array}{c} \Delta \phi \\ \Delta H\gamma \\ \alpha \end{array}\right]$$

Six equations are redundant for the solution of three unknown variables, so Δ*ϕ*, Δ*H_γ_* and *α* can be estimated by using the least-square method [45].

(27) $$\left[\begin{array}{c} \Delta \hat{\phi }\\ \Delta {Ĥ}_{\gamma }\\ \hat{\alpha } \end{array}\right]={\left\{{\left[\begin{array}{c} {\text{C}}_{1}\\ {\text{C}}_{2} \end{array}\right]}^{T}\cdot \left[\begin{array}{c} {\text{C}}_{1}\\ {\text{C}}_{2} \end{array}\right]\right\}}^{-1}\cdot \left[\begin{array}{c} {\text{C}}_{1}\\ {\text{C}}_{2} \end{array}\right]\cdot \left[\begin{array}{c} {\text{Z}}_{1}\\ {\text{Z}}_{2} \end{array}\right]$$

where, the parameters with the subscription “∧” denote the estimates. When there are more than two reference points available, the joint observation equation can be extended on the foundation of Equation (25). For example, for the condition of three reference points, the joint observation equation can be expressed as:

(28) $$\left[\begin{array}{c} \Delta \hat{\phi }\\ \Delta {Ĥ}_{\gamma }\\ \hat{\alpha } \end{array}\right]={\left\{{\left[\begin{array}{c} {\text{C}}_{1}\\ {\text{C}}_{2}\\ \text{C}3 \end{array}\right]}^{T}\cdot \left[\begin{array}{c} {\text{C}}_{1}\\ {\text{C}}_{2}\\ \text{C}3 \end{array}\right]\right\}}^{-1}\cdot \left[\begin{array}{c} {\text{C}}_{1}\\ {\text{C}}_{2}\\ \text{C}3 \end{array}\right]\cdot \left[\begin{array}{c} {\text{Z}}_{1}\\ \begin{array}{l} {\text{Z}}_{2}\\ {\text{Z}}_{3} \end{array} \end{array}\right]$$

After the estimation, the estimated results Δ*ϕ̂*, Δ*Ĥ_γ_* and *α̂* can be used to calculate the coordinate modification ${\left[\begin{array}{ccc} \Delta {\hat{x}}_{T}^{e}, & \Delta {\hat{y}}_{T}^{e}, & \Delta {\hat{z}}_{T}^{e} \end{array}\right]}^{T}$ by Equation (24). Then, the coordinate value of the target can be compensated, and the accurate coordinate of target can be calculated as follows.

(29) $$\left[\begin{array}{c} {\hat{x}}_{T}^{e}\\ {\hat{y}}_{T}^{e}\\ {\hat{z}}_{T}^{e} \end{array}\right]=\left[\begin{array}{c} {\tilde{x}}_{T}^{e}\\ {ỹ}_{T}^{e}\\ {\tilde{z}}_{T}^{e} \end{array}\right]-\left[\begin{array}{c} \Delta {\hat{x}}_{T}^{e}\\ \Delta {\hat{y}}_{T}^{e}\\ \Delta {\hat{z}}_{T}^{e} \end{array}\right]$$

It shows that both the installation and attitude errors can be simultaneously estimated by implementing the calibration algorithm, and this method could provide more accurate 3D position of target.

## 5. Simulation

### 5.1. Simulation for the Calibration Performance

**Figure 4.**
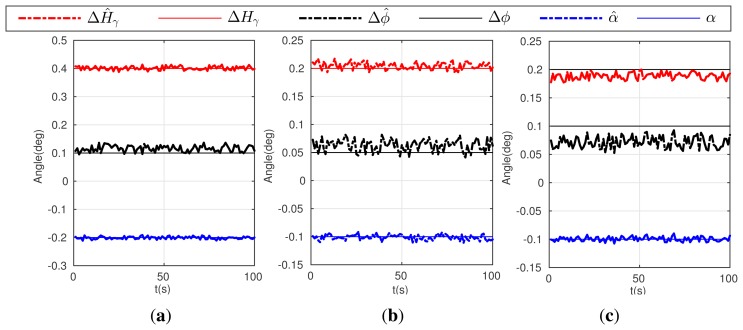
Estimation result for the calibration algorithm and the actual error: (**a**) curves on Condition 5; (**b**) curves on Condition 6; (**c**) curves on Condition 7.

**Figure 5.**
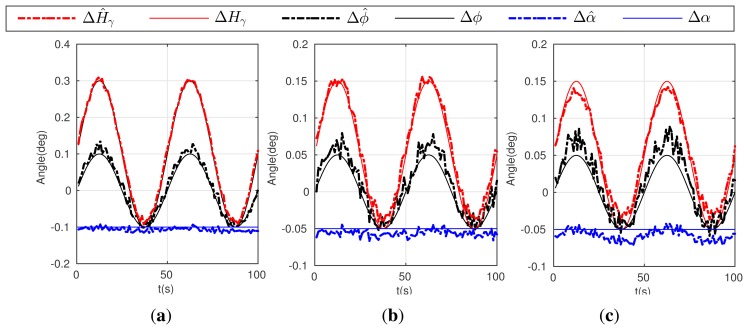
Estimation result for the calibration algorithm and the actual error. (**a**) curves on Condition 8; (**b**) curves on Condition 9; (**c**) curves on Condition 10.

In order to access the calibration performance, simulations are conducted with various conditions. During the simulation, the initial position, the positioning error of INS/GPS and the LDS’s error are the same as in Section 3. Related six conditions are set as following:

Condition 5: *α* = *γ* = −0.2°, *d_oR_*_1_ = 300 m, *d_oR_*_2_ = 280 m, Δ*ϕ* = 0.1°, Δ*H* = 0.2°;
  where, *d_oR_*_1_ denotes the distances between the point *o* and the reference point *R*_1_**, and *d_oR_*_2_ for *R*_2_.
Condition 6: *α* = *γ* = −0.1°, *d_oR_*_1_ = 300 m, *d_oR_*_2_ = 280 m, Δ*ϕ* = 0.05°, Δ*H* = 0.1°;
Condition 7: *α* = *γ* = −0.1°, *d*_o_*_R_*_1_ = 200 m, *d_oR_*_2_ = 140 m, Δ*ϕ* = 0.05°, Δ*H* = 0.1°;
  Figure 4 gives the simulation result of the first three conditions above (5,6,7).
Condition 8: *α* = *γ* = −0.2°, *d_oR_*_1_ = 300 m, *d_oR_*_2_ = 280 m, Δ*H* = 0.2° sin(2*π* × *t*/50), Δ*ϕ* = 0.1° sin(2*π* × *t*/50);
Condition 9: *α* = *γ* = −0.1°, *d_oR_*_1_ = 300 m, *d_oR_*_2_ = 280 m, Δ*H* = 0.1° sin(2*π* × *t*/50), Δ*ϕ* = 0.05° sin(2*π* × *t*/50);
Condition 10: *α* = *γ* = −0.1°, *d_oR_*_1_ = 200 m, *d_oR_*_2_ = 140 m, Δ*H* = 0.1° sin(2*π* × *t*/50), Δ*ϕ* = 0.05° sin(2*π* × *t*/50);

Figure 5 gives the simulation result of the later three conditions (8–10).

As shown in Figure 4a,b, although condition 5 and 6 give different error values, the calibration algorithm can realize accurate estimations. In comparing Figure 4b with 4c, the calibration accuracy decreases slightly, especially for the estimates of Δ*ϕ̂* and Δ*Ĥ_γ_*, when the reference points are much closer to observer. In addition, in the condition of varying errors, the proposed calibration algorithm can also estimate the types of errors given in condition 8, 9 and 10, which shows similar estimate performance seen in Figure 5a–c. Simulation results can prove that the calibration algorithm have a good performance in estimating both the installation and attitude measure error.

### 5.2. Simulation for Positioning Performance after Calibration

Simulations above demonstrates the calibration algorithm can realize simultaneous estimation of errors. Consequently, these estimated errors can be used for compensation, which would be very helpful for improving the positioning accuracy of target. Simulations under another three conditions are considered to evaluate the positioning performance of the scheme after simultaneously calibration.

**Figure 6.**
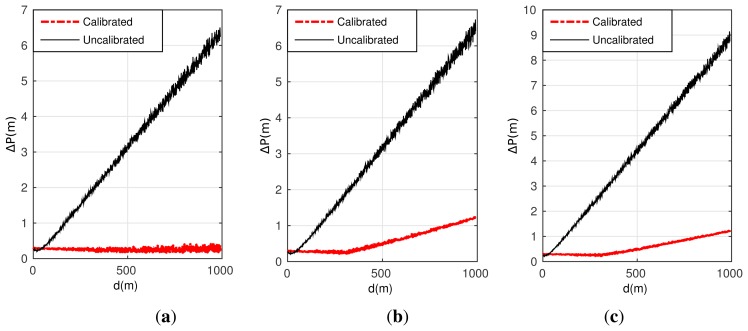
Estimation result for the calibration algorithm and the actual error. (**a**) curves on Condition 11; (**b**) curves on Condition 12; (**c**) curves on Condition 13.

Condition 11: *α* = *γ* = −0.2°, *d_oR_*_1_ = 300 m, *d_oR_*_2_ = 280 m, Δ*ϕ =* 0.1°, Δ*H* = 0.2°; without considering the measurement error and noise of LDS;
Condition 12: *α* = *γ* = −0.2°, *d_oR_*_1_ = 300 m, *d_oR_*_2_ = 280 m, Δ*ϕ* = 0.1°, Δ*H* = 0.2°;
Condition 13: *α* = *γ* = −0.1°, *d_oR_*_1_ = 200 m, *d_oR_*_2_ = 140 m, Δ*ϕ* = 0.1°, Δ*H* = 0.2°;

Figure 6 gives the positioning performance comparison between the un-calibrated and calibrated approaches corresponding to these three conditions.

As shown in Figure 6, it is obvious that the position errors without calibration are much larger than the schemes adopt the designed calibration algorithm. When the measurement errors of LDS are not involved, the positioning error is close to zero at the distance of 1 km after well calibration, as seen in Figure 6a. Even when the measurement errors of LDS have been considered, the corresponding positioning errors in Figure 6b,c are accumulated but still less than 2 m at the distance of 1 km. As a result, the positioning approach based on INS/GPS/LDS and the simultaneously designed calibration algorithm gain an improvement on the positioning accuracy, and are extremely efficient for a long range of 1 km.

## 6. Conclusions

In this paper, we present a simultaneously calibration approach for an INS/GPS/LDS Target Tracker to compensate the installation and attitude Errors. This approach is based on the attitude and position measured by INS/GPS system, and the distance between observer and target measured by LDS. The installation error, which exists between INS/GPS and LDS subsystems, are focused and researched. Its influence on positioning performance is investigated. Then, a simultaneous calibration algorithm is designed to estimate both the installation error and the attitude error of INS/GPS. Finally, simulations are conducted to demonstrate the validity of the proposed calibration algorithm as well as the entire target positioning method.

As the following work, in the realization of the prototype, the low-cost Micro-electro-mechanical systems (MEMS) based INS could also be applied into this kind of system. In its corresponding calibration method, the property of MEMS/GPS/LDS should be analyzed. On the other hand, in the real test, the level-arm effect caused by introducing reference points should be considered.
